# The association between sleep quality and loneliness in rural older individuals: a cross-sectional study in Shandong Province, China

**DOI:** 10.1186/s12877-020-01554-3

**Published:** 2020-05-24

**Authors:** Gaizhen Jia, Ping Yuan

**Affiliations:** Department of Epidemiology and Statistics, West China School of Public Health and West China Fourth Hospital, Sichuan University, No. 17,3 section South Renmin Road, Chengdu, 610041 Sichuan China

**Keywords:** Rural older adults, Loneliness, Sleep quality, Profile analysis

## Abstract

**Background:**

There is a evidence of negative association between loneliness and sleep quality in older adults. However, little is known regarding the relationship between loneliness and sleep quality among Chinese rural older adults. This study examined the associations of loneliness and sleep quality in a cross-sectional study of older adults.

**Methods:**

A face-to-face questionnaire survey was conducted among 1658 rural older adults in Shandong Province, China. Loneliness was assessed using the University of California at Los Angeles Loneliness Scale. Sleep quality was assessed using the Pittsburgh Sleep Quality Index. Ordinal logistic regression was conducted to examine the association of loneliness and sleep quality after adjustment for multiple confounding variables.

**Results:**

After variables such as age, marriage, education, occupation, economic income, family relationships, living arrangement, smoking behavior, alcohol consumption, chronic disease experience, and quality of life were controlled in a multivariable analysis, poor sleep quality was still associated with loneliness in the rural older population.

**Conclusion:**

This finding implied an adverse effect of sleep quality on the loneliness of older adults. Poor sleep quality was associated with increased odds of loneliness in Chinese rural older adults. Sleep-based interventions should be developed to prevent loneliness in rural older adults in China.

## Background

Loneliness experience is an important aspect of the mental health of older individuals [1]. Loneliness is also an important indicator for measuring the subjective well-being of the older population [2]. Surveys have shown that loneliness has become one of the prominent problems threatening the quality of life and personal happiness of older individuals [3].

The aging process of China’s population is obviously faster than that of other middle- and low-income countries. By 2040, the proportion of people aged 60 and over will rise to 28% from 12.4% in 2010. The life expectancy of women is higher than that of men, and the proportion of older people in rural areas is higher than that in urban areas (2016 National Assessment Report on Aging and Health in China).

According to a survey conducted by the China Aging Science Research Center, more than 1/3 of the older population in rural areas often feel severely lonely. In particular, the psychological impact of having an “empty nest” on older adults and the psychological problems of this population need to be addressed and solved [4, 5]. Peplau et al. began to study the loneliness of adults in the 1970s [6]. They proposed that loneliness is a negative emotion experienced by an individual when his or her social network is insufficient, including in terms of the inadequacy, low quality and low quantity of their social relationships [7, 8].

In the past 10 years, American scholar Dr. Cacioppo and his colleagues have conducted research on loneliness. They proposed an evolutionary theory of loneliness (ETL) that proposes that an individual’s genes determine his or her loneliness level [9]. Dr. Cacioppo also suggested that loneliness spreads similarly to an infectious disease [10]. The reported prevalence of loneliness in the older Chinese population varies greatly due to differences in research design, sample size, measurement of loneliness, regional economic and health threats and so on [4, 11].

Many factors have been indicated to be associated with loneliness. These factors include demographic characteristics, such as gender, age, education level [12], marital and economic status, social interaction [13, 14], race [15], experience of stress or depression [16, 17], poor lifestyle [18], single relationship status, malnutrition, relationships with family members [19], life satisfaction [20], alcohol consumption [21], smoking behavior, quality of life, noncommunicable disease (NCD) experience [22], and experience of psychological problems [23]. However, these factors, including gender, age, marriage, race, chronic disease experience and other demographic characteristics, are often difficult or impossible to change, so it is undoubtedly important to study factors that can be easily targeted by interventions, such as sleep, life satisfaction, and nutrition, to improve the loneliness of aging adults.

Loneliness is associated with poor health outcomes, including depression, sleep quality, heart disease and all-cause mortality [24]. Among the psychological and behavioral problems experienced by lonely people, poor sleep quality has received considerable attention in recent years [25]. Studies have suggested that loneliness causes poor sleep quality and insomnia. In the ETL, Cacioppo [26] proposed that loneliness leads to poor sleep quality and insomnia (and other results) because it signals that one exists in an environment where others are likely to behave selfishly. This signal triggers a physiological response to the perceived possibility of a social threat (even if there is no threat), including increased cortisol secretion, which is a common substance secreted when one is awake that can have a negative impact on sleep initiation and that is not conducive to high-quality sleep [27]. This theory was supported by Matthews [28], who found that the link between loneliness and sleep quality was particularly significant in young people who had experienced trauma, such as violence/neglect.

Loneliness and poor sleep quality are common phenomena in advanced age and are not conducive to physical and mental health. Although it may be difficult to provide effective interventions for loneliness, there may be some related adjustable factors, such as sleep. If sleep disorders are relieved, the impact of loneliness on the health of the older population can be reduced. McHugh [29] found that the effect of loneliness, especially emotional loneliness, on the sleep quality of older individuals was partly mediated by perceived stress. The research results on loneliness and sleep quality in the older population are mixed. A study with a large sample of older adults in China showed that compared with older individuals with an empty nest, older individuals who lived with their spouse and children together or family members had better sleep quality [30]. The study also found that loneliness was associated with a decline in the subjective sleep quality of older individuals [31]. Another study reported that loneliness did not affect sleep duration or subjective sleep quality [32]. The reasons for the inconsistency in the research on the relationship between sleep problems and loneliness may be related to the participants’ national background, the research population, a small sample size, the use of different evaluation tools for loneliness and sleep, and the absence of analysis of socioeconomic factors that affect loneliness. Therefore, it is necessary to select a larger sample size and measurement tools with greater validity to study the relationship between sleep problems and loneliness under the control of socioeconomic and other factors. Wakefield’s research suggested that loneliness is an important predictor of depression and sleep deprivation. Conversely, sleep problems may be an antecedent of loneliness [33]. However, the prediction of loneliness based on sleep has not been determined, and other variables that may predict loneliness have not been fully studied [27].

A study by Chris Segrin conducted among 255 couples in the United States indicated an association between loneliness and sleep quality [25]. The relationship between loneliness and sleep quality was also explored among American college students [24]. Another study showed that loneliness in young adults could affect their sleep quality [28]. However, most of the above studies focused on young people or college students.

A survey of 447 older individuals in an urban Irish community showed an effect of emotional loneliness on the quality of sleep, but the study used the De Jong Gierveld Loneliness Scale with a small sample size [29]. An ongoing longitudinal study of a sample in Taiwan has not revealed an association between loneliness and sleep quality in older people [34]. In view of the uncertainty of the above findings and the frequent use of small sample sizes, it is necessary to conduct a study with a large sample on the relationship between loneliness and sleep quality.

Therefore, we recruited a large sample of people from rural areas in Shandong Province, China, to study the association between loneliness and sleep quality in the older population. To this end, we established the following specific objectives. First, we aimed to identify the prevalence of loneliness among the older population in Shandong Province, China. Second, we studied the relationship between loneliness and sleep quality in the older population.

## Methods

### Settings and participants

A village-based cross-sectional study was conducted among 1658 older.

people over 60 years of age in five cities in Shandong Province. The participants were selected by three-stage cluster sampling. First, according to the geographical location of Shandong Province, each district and county was divided into five groups: east, south, west, north and central. Second, we chose one city from each of the five groups: Yantai (east), Jining (south), Liaocheng (west), Binzhou (north) and Zibo (central). Similarly, four towns were sampled from each sample city according to their geographical locations. Finally, one village was sampled from each town, and all older individuals in these 20 villages were included in this study. The average number of people in each village was 90, the response rate was 94.27%, and the effective rate was 97.82%.

### Data collection

The entire survey was conducted from December 2016 to February 2017. All of the older adults were interviewed face to face by trained investigators who were undergraduates of Binzhou Medical University School of Public Health and Management. Information on demographics, loneliness, sleep quality, quality of life, health-related behaviors, diseases and symptoms was collected with a structured questionnaire. To ensure quality, inspectors checked each completed questionnaire daily and sought supplementary information and corrected errors in a timely manner.

### Measures

#### Sociodemographic variables

Sociodemographic characteristics, such as sex, age, economic status (source and quantity of economic income), marital status, education, and occupation, were investigated. The ages of the participants were categorized as follows: 60–69, 70–79 and 80+ years old. Other demographic characteristics were classified as follows: sex (male vs. female), quantity of economic income (< 1000 yuan, 1000–3000 yuan, 3000–5000 yuan, and ≥ 5000 yuan), source of economic income (farming income vs. support from children and others), marital status (couple vs. other), education (primary school or below, junior school, and senior high school and above), occupation (manual labor vs. mental labor), number of children (0, 1, 2, and 3+), relationship with family members (good vs. bad), and empty nester (yes vs. no).

#### Health-related behaviors

Smoking behavior and alcohol consumption were assessed by asking the participants whether they had smoked or consumed alcohol in the past 6 months [35].

#### Diseases and symptoms

Self-reported noncommunicable diseases (NCDs) were also investigated, especially NCDs in the past year (yes vs. no). Weight and height were measured in this study, and body mass index (BMI) was calculated as weight (kg) divided by height (m) divided by height (m) again. Participants were divided into 3 categories (lean, normal and overweight+) according to the Chinese standard.

#### Quality of life scale

The Medical Outcomes Study 36-item Short Form Health Survey (SF-36) is a generic tool for assessing health-related quality of life [36]. The results are evaluated through the assignment of scores for each question and then the transformation of the scores based on a scale from 0 to 100, with a higher score reflecting better perceived health [37]. The Cronbach’s α is 0.825 in this study.

#### Loneliness assessment

The University of California at Los Angeles (UCLA) Loneliness Scale (version 3) compiled by Russell et al. was administered to all groups [38]. The advantage of the third version of the scale is the reverse-scored items, which prevent false responses. The UCLA Loneliness Scale contains 20 items, and each item has a 4-point rating scale in which 1 = never and 4 = always. The item scores are summed to produce a total score, with potential scores ranging from 20 to 80. A higher score indicates a higher level of perceived loneliness. The scale is the most commonly used tool for assessing loneliness [39, 40]; the Cronbach’s α was 0.860 in this study. According to Perry’s loneliness classification scheme [41], a score of 20–34 is classified as a low level of loneliness experience, 35–49 is classified as a moderate level of loneliness experience, and a score of over 50 is classified as a high level of loneliness experience. The test-retest reliability of the scale is relatively high, which makes it suitable to measure loneliness in a sample of older adults [42].

#### Sleep quality assessment

We used the Pittsburgh Sleep Quality Index (PSQI), which is used worldwide, to evaluate sleep quality in the sample of older adults [43]. The scale includes the following 7 dimensions: subjective sleep quality, sleep latency, sleep duration, habitual sleep efficiency, sleep disturbances, use of sleeping medication, and daytime dysfunction [44]. Each dimension score ranges from 0 to 3 points. The sum of the 7 dimension scores is the total score of the PSQI (range of 0–21) [xir45]. The Cronbach’s α of the PSQI in this study was 0.840. In Li J’s research on the analysis of sleep quality characteristics and related factors in the older population in rural China, when 7 was used as the cutoff, the sensitivity and specificity were 98.3 and 90.2%, respectively [45]. The threshold for poor sleep quality was seven, with higher scores indicating poorer sleep quality [46].

### Statistical analysis

EpiData version 3.1 (The EpiData Association, Odense, Denmark) was used for data entry and verification. SPSS version 22.0 (IBM Corp., Armonk, NY) statistical software was used for statistical analysis. The differences in the loneliness scores by sociodemographic, health, quality of life and sleep quality factors were examined using independent sample *t*-tests or analysis of variance (ANOVA). An ordinal logistic regression model was employed to assess the association between sleep quality and loneliness. Three models were fitted for the outcome. An adjusted model (Model 1) was constructed first to examine the effects of different demographic variables on loneliness. A fully adjusted model (Model 2) was constructed to examine the associations between sleep quality and loneliness with sociodemographic variables, health-related behaviors, quality of life variables and NCDs controlled. Model 3 was constructed through the addition of all seven dimensions of sleep quality and loneliness to the fully adjusted model. A sensitivity analysis was conducted in which participants with 1 or 0 children were excluded. Leanness or obesity were considered to be the main risk factors for poor sleep quality, which can lead to poor quality of life. Therefore, subjects with BMI ≥ 24 or BMI < 18.5 were excluded from the second sensitivity analysis, and those with poor quality of life were excluded from the third sensitivity analysis. All reported CIs were calculated at the 95% level. Statistical significance was assessed at the 5% level.

## Results

### Basic information of the participants

Table 1 shows the basic information collected from the 1658 participants. The average age of the respondents was 70.41 years old (SD 7.63). The loneliness scores in the survey ranged between 21 and 80. A total of 31 (18.4%), 924 (55.7%), and 429 (25.9%) of the respondents were assessed as having low, moderate, and high levels of loneliness, respectively, and the mean overall score of the participants was 43.17 ± 9.46. Of all the participants, 50.7% were female, and 15.2% were over 80 years of age. Individuals who were in a couple relationship accounted for 69.8% of the respondents, and 31.1% of the participants reported less than 1000 yuan in economic income. A total of 62.1% of the participants reported that their income came from their children; 69.1% had a primary school education or below, and 63.0% worked in manual labor. In total, 30.9% reported smoking, and 46.1% reported consuming alcohol. In addition, 94.7% had good relationships with their family members, 30.2% reported being empty nesters, and 17.3% reported having either 1 child or 0 children. A total of 32.8% reported having a chronic disease.

**Table 1:** Comparison of Loneliness Scores among Rural Elderly with Different Characteristics (Mean ± SD)

Variables	N(%)	Loneliness score	F/t	P
Sex:				
Male	818 (49.3)	42.81 ± 9.54	−1.531	0.126
Female	840 (50.7)	43.52 ± 9.37		
Age:				
60 ~ 69	840 (50.7)	42.24 ± 9.47	10.781	0.000
70 ~ 79	566 (34.1)	43.64 ± 9.50		
80 + years older	252 (15.2)	45.21 ± 8.94		
Marital status:				
Couple	1158 (69.8)	42.65 ± 9.40	−3.431	0.001
Others^a^	500 (30.2)	44.38 ± 9.49		
Occupation:				
Manual labour	1045 (63.0)	41.37 ± 9.34	−10.588	0.000
Mental labour	613 (37.0)	46.24 ± 8.86		
Degree of education:				
Primary school or below	1145 (69.1)	42.06 ± 9.48	26.500	0.000
Junior school	266 (16.0)	45.82 ± 9.13		
High school and above	247 (14.9)	45.47 ± 8.72		
Economic sources:				
Farming income	628 (37.9)	40.44 ± 9.63	−9.244	0.000
Support from children and others	1030 (62.1)	44.83 ± 8.96		
Economic income (yuan):				
< 1000	516 (31.1)	42.66 ± 9.54	4.852	0.002
1000 ~ 3000	401 (24.2)	42.71 ± 9.00		
3000 ~ 5000	415 (25.0)	42.86 ± 9.42		
≥ 5000	326 (19.7)	44.95 ± 9.75		
Smoking:				
Yes	512 (30.9)	41.92 ± 9.88	−3.515	0.000
No	1146 (69.1)	43.73 ± 9.21		
Drinking:				
Yes	765 (46.1)	42.14 ± 9.45	−4.134	0.000
No	893 (53.9)	44.05 ± 9.38		
Relationship with family members:				
Good	1570 (94.7)	43.06 ± 9.42	−2.007	0.045
Bad	88(5.3)	45.14 ± 9.89		
Empty nester:				
Yes	501 (30.2)	45.77 ± 8.91	56.299	0.000
No	1157 (69.8)	42.04 ± 9.47		
Number of children:				
0	36(2.2)	44.64 ± 7.45	17.162	0.000
1	251 (15.1)	46.76 ± 9.08		
2	453 (27.3)	43.34 ± 9.58		
3+	918 (55.4)	42.05 ± 9.32		
BMI:				
Lean	146(8.8)	42.95 ± 9.57	1.980	0.138
Normal	776 (46.8)	42.73 ± 9.63		
Overweight+	736 (44.4)	43.68 ± 9.22		
NCDs:				
Yes	668 (32.8)	44.80 ± 8.93	33.769	0.000
No	990 (67.2)	42.07 ± 9.65		

^a^ others means those who are unmarried (1.7%), divorced (6.5%), widowed (22.0%)

### Comparison of loneliness scores

There were significant differences in loneliness scores among older adults with different characteristics, mainly including age (*p <* 0.001), marital status (*p* = 0.001), occupation (*p <* 0.001), education (*p <* 0.001), source of economic income (*p <* 0.001), quantity of economic income (*p* = 0.002), smoking behavior (*p <* 0.001), alcohol consumption (*p <* 0.001), relationships with family members (*p* = 0.045), empty nester status (*p <* 0.001), number of children (*p <* 0.001) and NCD experience (*p <* 0.001), as shown in Table 1. The average loneliness score of older individuals with poor sleep quality was 45.04 ± 8.86, which was significantly higher than that of older individuals with good sleep quality (41.66 ± 9.65) (*t* = 7.742, *p <* 0.001).

### Comparison of PSQI total and dimension scores

The mean sleep quality score was 6.67 ± 3.42. Among the 1658 rural older people in Shandong Province with global scores greater than 7, which accounted for 44.9% of the sample, the prevalence rate of poor sleep quality was 44.9%. As shown in Table 2, the average scores for sleep quality, subjective sleep quality, sleep latency, sleep duration, habitual sleep efficiency, sleep disturbances, use of sleeping medication, and daytime dysfunction were 6.67 ± 3.42, 1.14 ± 0.76, 1.24 ± 0.90, 0.61 ± 0.84, 0.84 ± 1.04, 1.34 ± 0.67, 0.36 ± 0.76 and 1.15 ± 0.78, respectively. There were statistically significant differences in the total sleep quality scores and 5 of the PSQI dimensions scores (all except subjective sleep quality and habitual sleep efficiency) among participants with different levels of loneliness (*p* < 0.05).

**Table 2:** Comparison of the PSQI and its component scores with different levels of loneliness in Shandong, China (2016) (mean ± s)

Variables	Total score	Different levels of loneliness			F	P
		Low	Moderate	High		
**Total PSQI**	6.67 ± 3.42	5.36 ± 2.83	6.69 ± 3.23	7.55 ± 3.89	38.104	0.000
**Components of PSQI**						
Subjective sleep quality	1.14 ± 0.76	1.06 ± 0.64	1.17 ± 0.72	1.11 ± 0.91	2.680	0.069
Sleep latency	1.24 ± 0.90	0.90 ± 0.83	1.23 ± 0.91	1.48 ± 0.86	38.583	0.000
Sleep duration	0.61 ± 0.84	0.30 ± 0.62	0.58 ± 0.83	0.88 ± 0.91	45.337	0.000
Habitual sleep efficiency	0.84 ± 1.04	0.78 ± 1.01	0.85 ± 1.05	0.87 ± 1.05	0.840	0.432
Sleep disturbances	1.34 ± 0.67	1.21 ± 0.50	1.36 ± 0.61	1.38 ± 0.86	6.667	0.001
Use of sleeping medication	0.36 ± 0.76	0.17 ± 0.52	0.32 ± 0.69	0.59 ± 0.96	32.302	0.000
Daytime dysfunction	1.15 ± 0.78	0.94 ± 0.63	1.17 ± 0.71	1.23 ± 0.99	13.317	0.000

### Contour analysis of the average PSQI scores of participants with different levels of loneliness

**Fig. 1:**
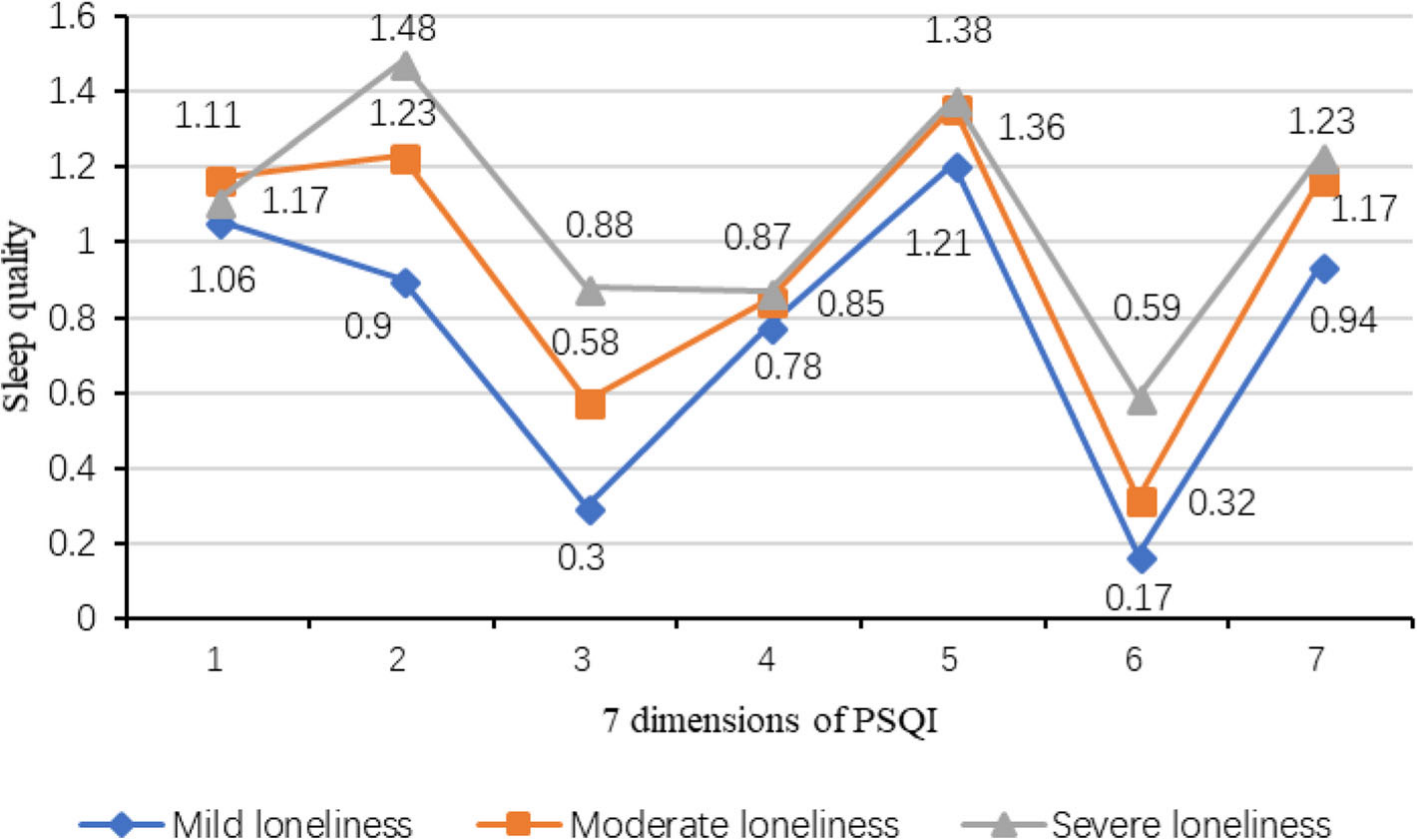
Profile chart of mean of the PSQI scores for different levels of loneliness in rural elderly people

A contour analysis of the average PSQI total scores of rural older people with different levels of loneliness was conducted. The profiles of older people in rural areas who experienced low, moderate, and high levels of loneliness were not parallel to each other (*F* = 12.000, *p* = 0.000), and the contours of the high group were higher than those of the moderate and low groups. In the horizontal profile analysis, the average scores for the 7 dimensions, namely, subjective sleep quality, sleep latency, sleep duration, habitual sleep efficiency, sleep disturbances, use of sleeping medication and daytime dysfunction, were different (*F* = 38.103, *p* = 0.000), as shown in Fig. 1.

### Association between sleep quality and loneliness

The level of loneliness in the rural older population was used as the dependent variable (Y = 0, low level of loneliness; Y = 1, moderate level of loneliness; and Y = 2, high level of loneliness). The sleep quality score was used as the independent variable, and age, marital status, occupation, source and quantity of economic income, relationships with family members, empty nester status, number of children, smoking behavior, alcohol consumption, BMI, chronic disease experience and quality of life were used as the control variables. The relationship between the sleep quality and loneliness of older people was analyzed by the three ordinal regression models. Even after marital status, alcohol consumption, relationships with family members, occupation, source and quantity of economic income, chronic disease experience and quality of life were controlled, an increase in the odds of loneliness was associated with an increase in the sleep quality score (adjusted odds ratio [aOR] = 1.111, 95% confidence interval [95% CI] = 1.078–1.145). The worse the quality of sleep was, the higher the degree of loneliness in the older adult sample. Scores for subjective sleep quality (aOR = 0.765, 95% CI = 0.649–0.902), sleep latency (aOR = 1.346, 95% CI = 1.178–1.537), sleep duration (aOR = 1.316, 95% CI = 1.139–1.522), use of sleeping medication (aOR = 1.175, 95% CI = 1.005–1.372), and daytime dysfunction (aOR = 1.267, 95% CI = 1.079–1.487) were significantly different between older adults with high levels of loneliness and those with moderate and low levels of loneliness. Older adults with higher PSQI scores for these 5 dimensions had a higher risk of loneliness. Although older adults with higher scores for habitual sleep efficiency and sleep disturbances had a higher risk of loneliness, these differences were not statistically significant, as shown in Table 3.

**Table 3:** Associations between sleep quality and loneliness in the rural elderly individuals in Shandong, China (2016)

Variables	Loneliness					
	Model 1		Model 2		Model 3	
	***OR(*95%CI)**	***p***	***OR(*95%CI)**	***p***	***OR(*95%CI)**	***p***
Age:						
60’s	1		1		1	
70’s	1.950 (1.448–2.627)	0.000	1.465 (1.167–1.839)	0.013	1.443 (1.148–1.815)	0.002
80+	1.470 (0.172–1.840)	0.001	1.866 (1.382–2.519)	0.000	1.795 (1.324–2.433)	0.000
Sex:						
Female	1		1		1	
Male	0.885 (0.722–1.085)	0.241	0.898 (0.732–1.102)	0.304	0.904 (0.736–1.112)	0.340
Marital status:						
Others^a^	1		1		1	
Couple	1.148 (0.917–1.438)	0.231	1.191 (0.949–1.495)	0.131	1.172 (0.933–1.473)	0.172
Degree of education:						
Primary school or below	1		1		1	
Junior school	1.752 (1.306–2.351)	0.000	1.749 (1.300–2.351)	0.000	1.664 (1.234–2.243)	0.001
High school and above	0.937 (0.666–1.319)	0.901	1.040 (0.638–1.271)	0.552	0.876 (0.620–1.239)	0.455
Occupation:						
Mental labour	1		1		1	
Manual labour	0.478 (0.369–0.619)	0.000	0.491 (0.379–0.637)	0.000	0.552 (0.422–0.722)	0.000
Economic income:(RMB)						
less than 1000	1		1		1	
1000 ~ 3000	1.169 (0.981–1.786)	0.245	1.220 (0.937–1.589)	0.140	1.225 (0.939–1.598)	0.134
3000 ~ 5000	0.923 (0.701–1.214)	0.565	0.980 (0.744–1.292)	0.887	0.982 (0.744–1.296)	0.897
5000and above	1.324 (0.981–1.786)	0.066	1.201 (0.890–1.632)	0.226	1.115 (0.820–1.517)	0.488
Family relationship:						
Good	1		1		1	
Bad	1.517 (0.987–2.333)	0.058	1.543 (1.002–2.425)	0.049	1.547 (1.001–2.387)	0.049
Empty nester:						
No	1					
Yes	1.519 (1.207 ~ 1.912)	0.000	1.553 (1.231 ~ 1.958)	0.000	1.514 (1.198 ~ 1.912)	0.000
Number of children:						
3+	1					
2	1.132 (0.895 ~ 1.433)	0.301	1.126 (0.890 ~ 1.428)	0.322	1.065 (0.839 ~ 1.351)	0.607
1	1.950 (1.420 ~ 2.678)	0.000	1.946 (1.415 ~ 2.680)	0.000	1.833 (1.327 ~ 2.529)	0.000
0	1.235 (0.628 ~ 2.428)	0.540	1.150 (0.583 ~ 2.270)	0.687	1.041 (0.525 ~ 2.063)	0.910
Smoking:						
NO	1		1		1	
Yes	0.746 (0.592–0.939)	0.013	0.855 (0.676–1.081)	0.190	0.890 (0.715–1.131)	0.342
Drinking:						
NO	1		1		1	
Yes	0.992 (0.803–1.228)	0.944	0.949 (0.766–1.176)	0.634	1.022 (0.819–1.276)	0.847
BMI:						
Normal	1		1		1	
Lean	1.206 (0.849–1.714)	0.295	1.212 (0.850–1.725)	0.288	1.158 (0.811–1.652)	0.418
Overweight+	1.140 (0.930–1.398)	0.208	1.078 (0.877–1.324)	0.474	1.059 (0.861–1.302)	0.588
NCDs:						
No	1		1		1	
Yes	1.319 (1.079–1.613)	0.007	1.270 (1.037–1.556)	0.021	1.287 (1.048–1.579)	0.016
Quality of life	1.006 (1.001–0.010)	0.009	1.007 (1.002–1.011)	0.002	1.007 (1.002–1.011)	0.003
Sleep quality			1.111 (1.078–1.145)	0.000		
Subjective sleep quality					0.765 (0.649–0.902)	0.001
Sleep latency					1.346 (1.178–1.537)	0.000
Sleep duration					1.316 (1.139–1.522)	0.000
Habitual-sleep efficiency					1.350 (0.925–1.148)	0.588
Sleep disturbances					0.984 (0.811–1.194)	0.873
Use of sleeping medication					1.175 (1.005–1.372)	0.042
Daytime dysfunction					1.267 (1.079–1.487)	0.004

### Sensitivity analysis

We conducted a sensitivity analysis to further assess the robustness of the model and better explain the reliability of the results. A sensitivity analysis that excluded participants with either one child or no children (*n* = 287) yielded a similar result to that of Model 2 (OR 1.102; 95% CI 1.064–1.141; *p* = 0.000). The second sensitivity analysis that excluded participants with a BMI ≥ 24 or a BMI < 18.5 (*n* = 882) also yielded a result similar to that of Model 2 (OR 1.113; 95% CI 1.063–1.164; *p* = 0.000). The third sensitivity analysis that excluded participants with poor quality of life (*n* = 160) also yielded a result similar to that of Model 2 (OR 1.105; 95% CI 1.070–1.141; *p* = 0.000).

## Discussion

The overall loneliness score of rural older adults from Shandong Province rural was 43.17 ± 9.46, and 25.9% of the study sample had a strong sense of loneliness. This percentage is higher than the 11.6% reported for a population of English-speaking U.S. residents aged more than 65 years [20]. It is also higher than the 10.5% reported for the general population in Germany [23]. However, the percentage is lower than the 38.22% reported in a study of older individuals with similar ages in urban communities of Xiamen, China [47]. Differences in socioeconomic development might explain the variations between our study findings and those of studies carried out in the U.S. and Germany [46]. The acceleration of rural urbanization caused by socioeconomic development in China has led to a rapid increase in the number of empty nesters, and having an empty nest eventually leads to social isolation and an increase in psychological problems for rural older individuals who are not accompanied by their children. The difference between the results of our research and those of the study of urban Xiamen may be due to differences in the geographical location. Our research was conducted in rural Shandong Province, while the study in Xiamen was conducted in urban areas. Compared with older adults living in urban areas, older adults living in rural regions have better neighborhood relationships, which can meet the needs of older adults by providing opportunities for social activities and emotional contact to alleviate loneliness. However, due to retirement, older people in cities experience a loss of social relations. In addition, the prevalence of chronic diseases and the divorce rate of older adults in rural areas are lower than those in urban areas. Physical diseases limit older adults’ ability to participate in social activities, and the absence of spouses also reduces the social support of older adults, thus reducing social ties and leading to loneliness. In addition, the differences between our research and the Xiamen study can be explained by the use of different investigative tools. We used a universal scale to evaluate loneliness. However, in the Xiamen study, only 1 question was used to test loneliness.

The main factors affecting loneliness in this study were age, marriage, education, occupation, source and quantity of economic, family relations, empty nester status, number of children, smoking behavior, alcohol consumption, NCD experience and so on, which is consistent with the findings of previous studies [48]. Many studies have suggested that social and economic factors play an important role in the development of loneliness among older individuals in rural areas [49]. Among these factors, the empty nest phenomenon is the most obvious social factor in China’s rural areas. Since the 1980s, a large portion of the rural labor force has continuously migrated into the city, which intensifies the empty nest phenomenon among the rural older population. In this study, the level of loneliness of older empty nesters was significantly higher than that of older individuals who were not empty nesters, which is consistent with previous studies. Furthermore, chronic diseases leads to a decline in the health status of older individuals, which makes social communication difficult. Social support by children for older individuals decreases when children move away from home and their parents become empty nesters. The death of a spouse also reduces the social support of older individuals, further promoting the loneliness of the older population [50]. The influence of the number of children (a specifically added variable) on the loneliness of older individuals showed that having no children or only 1 child was associated with higher levels of loneliness than having 2 or more children, which further demonstrates the importance of the companionship of children for older individuals.

Research shows that deficiencies in the quantity and quality of sleep can predict some health problems [51, 52]. This is consistent with previous studies in different populations that have found a negative correlation between loneliness and sleep quality. Matthews et al. [28] found evidence of this correlation in young people, as did Wakefield [27] in a group of individuals aged 18–76 years old. Although the research populations were different, these findings are consistent with the ETL [26] tested by Matthews et al. Because loneliness promotes the perception of social threats, there is a negative correlation between loneliness and sleep quality. The perception of social threats in turn triggers physiological reactions, such as cortisol production, which can disrupt sleep attempts. This may be one of the reasons why loneliness is negatively related to sleep. McHugh [29] proposed that emotional loneliness in the older population may lead to an increase in perceived stress, which in turn affects sleep quality. Although some possible explanations have been provided for the relationship between loneliness and sleep, it seems to be easier and more effective to improve the quality of life of older individuals through sleep interventions than through efforts to improve loneliness.

This study attempted to explore whether loneliness affects the sleep quality of rural older people. To reduce the possibility of finding an association between loneliness and sleep caused by mixed bias, our study further excluded other factors related to loneliness. When covariates such as age, marital status, education, occupation, economic status, family relationships, lifestyle, smoking behavior, alcohol consumption, BMI, NCD experience and quality of life were controlled, the PSQI total score in the sample of older adults was higher, and the level of loneliness experience decreased [53]. Our results are consistent with those of studies by Matthews et al. on the relationship between sleep quality and loneliness in young people, suggesting that poor sleep quality increases the risk of loneliness even after many other factors are controlled [28, 32, 54, 55]. Our findings are not consistent with previous studies in older adults in the Taiwan area [34]. The difference between our study and the study of Yu B et al., in addition to geographical differences, may be related to the use of different survey tools to evaluate loneliness. In the research on Taiwan, loneliness was evaluated by only one question, whereas we used the UCLA Loneliness Scale to evaluate loneliness. Some physiological processes may also explain the association between loneliness and sleep quality. Dream disturbances are associated with greater stress and anxiety [56] and may represent a further manifestation of emotional distress in lonely individuals [28].

Our study also showed that 5 dimensions of sleep quality, including subjective sleep quality, sleep latency, sleep duration, use of sleeping medication, and daytime dysfunction, were associated with loneliness. In previous studies of loneliness and sleep in young adults, only subjective sleep quality and daytime dysfunction were associated with loneliness [28, 32]; however, the study of the older population in Taiwan did not explore the dimensions of the PSQI scale. A study of 95 young people showed that loneliness was related to sleep quality, but no relationship between loneliness and sleep duration was found [54]. Older people with poorer subjective sleep quality are more likely to become restless than are those with no subjective sleep quality [57]. Poor subjective sleep quality is harmful to a person’s physical and mental health. Studies have shown that physical and mental health are related to loneliness [18]. These negative effects may in turn promote increased loneliness. The influence of the dimensions of sleep quality on loneliness should be further studied.

Based on the findings in this sample, the relationship between sleep quality and loneliness is very strong in the older population in China. Previous studies have also shown a relationship between loneliness and sleep quality, indicating that loneliness affects sleep and that sleep also affects loneliness [55, 58]. The connection between the two is exacerbated by stress [25]. Whether there are intermediate variables between loneliness and sleep quality and how these intermediate variables function need to be explored in future studies; however, the relationship between the loneliness and sleep quality of the older population in rural areas is self-evident and should be important to decision makers. Psychological interventions to improve the loneliness of older adults or measures to improve their sleep quality would ultimately improve their quality of life.

Our study has some limitations that must be considered in the interpretation of the findings. First, loneliness and sleep quality were measured cross-sectionally, so no conclusions can be drawn about the directionality of the associations. There is not enough evidence to determine whether loneliness precedes sleep disorders or vice versa. Therefore, follow-up studies should be carried out in the future to demonstrate the direction of the causal relationship between sleep disorders and loneliness. Second, the assessment of both loneliness and sleep quality was based on self-reported measures that can cause reporting bias. Therefore, we suggest that direct and indirect loneliness measurement methods should be used in future work to explore their predictive validity in more detail. Regarding the evaluation of sleep quality, we also suggest that future research should focus on more objective sleep measurement methods (such as actigraphy, the use of actigraphy watches, and polysomnography) to further study the relationship between loneliness and sleep in older adults. Third, unknown variables were not included in the designed questionnaire, which may have led to some deviations in the results. For example, the weakening of the social communication ability of older adults is significant to their feeling of loneliness [59], but it was not included as a confounding factor in this study. The exclusion of such variables may have affected the validity of our findings.

## Conclusion

In summary, our study demonstrated that sleep quality was significantly associated with loneliness in the rural older population in China. We also found that several variables, such as subjective sleep quality, sleep latency, sleep duration, use of sleeping medication, and daytime dysfunction, were significantly different between participants with high levels of loneliness and those with moderate and low levels of loneliness. Interventions should be developed to help older individuals with sleep disorders improve their sleep quality so that high levels of loneliness are prevented in the older population in China. Therefore, corresponding intervention measures should be formulated to reduce the loneliness of older individuals and improve the quality of sleep in the older population.

## Data Availability

The datasets used and/or analyzed during the article are available on reasonable request to jiagzh221@163.com.

## Abbreviations

BMI: Body mass index
PSQI: Pittsburgh Sleep Quality Index
CI: Confidence interval
OR: Odds ratio
SD: Standard deviation
SPSS: Statistical Package for the Social Sciences
NCDs: Noncommunicable diseases
